# Prevalence of COVID-19 Diagnostic Output with Chest Computed Tomography: A Systematic Review and Meta-Analysis

**DOI:** 10.3390/diagnostics10121023

**Published:** 2020-11-28

**Authors:** Temitope Emmanuel Komolafe, John Agbo, Ebenezer Obaloluwa Olaniyi, Kayode Komolafe, Xiaodong Yang

**Affiliations:** 1 Division of Life Sciences and Medicine, School of Biomedical Engineering (Suzhou), University of Science and Technology of China, Hefei 230026, China; teakomo@mail.ustc.edu.cn; 2 Department of Medical Imaging, Suzhou Institute of Biomedical Engineering and Technology, Chinese Academy of Sciences, Suzhou 215163, China; 3 CAS Key Laboratory for Brain Function and Disease, Department of Neurobiology, University of Science and Technology of China, Hefei 230026, China; agbojbhn@mail.ustc.edu.cn; 4 Department of Neuropharmacology, Faculty of Basic Medical Sciences, Abubakar Tafawa Balewa University, Bauchi 740272, Nigeria; 5 Department of Biomedical Engineering, Shenzhen University, Shenzhen 518060, China; olaniyiebenezer@adelekeuniversity.edu.ng; 6 Department of Electrical and Electronic Engineering, Adeleke University, Ede 232103, Nigeria; 7 Department of Biochemistry, Federal University of Oye Ekiti, Oye 371104, Nigeria; kayode.komolafe@fuoye.edu.ng; 8 Jihua Laboratory, Foshan 528000, China

**Keywords:** age distribution, computed tomography, COVID-19, prevalence, meta-analysis

## Abstract

Background: The pooled prevalence of chest computed tomography (CT) abnormalities and other detailed analysis related to patients’ biodata like gender and different age groups have not been previously described for patients with coronavirus disease 2019 (COVID-19), thus necessitating this study. Objectives: To perform a meta-analysis to evaluate the diagnostic performance of chest CT, common CT morphological abnormalities, disease prevalence, biodata information, and gender prevalence of patients. Methods: Studies were identified by searching PubMed and Science Direct libraries from 1 January 2020 to 30 April 2020. Pooled CT positive rate of COVID-19 and RT-PCR, CT-imaging features, history of exposure, and biodata information were estimated using the quality effect (QE) model. Results: Out of 36 studies included, the sensitivity was 89% (95% CI: 80–96%) and 98% (95% CI: 90–100%) for chest CT and reverse transcription-polymerase chain reaction (RT-PCR), respectively. The pooled prevalence across lesion distribution were 72% (95% CI: 62–80%), 92% (95% CI: 84–97%) for lung lobe, 88% (95% CI: 81–93%) for patients with history of exposure, and 91% (95% CI: 85–96%) for patients with all categories of symptoms. Seventy-six percent (95% CI: 67–83%) had age distribution across four age groups, while the pooled prevalence was higher in the male with 54% (95% CI: 50–57%) and 46% (95% CI: 43–50%) in the female. Conclusions: The sensitivity of RT-PCR was higher than chest CT, and disease prevalence appears relatively higher in the elderly and males than children and females, respectively.

## 1. Introduction

Coronavirus disease 2019 (COVID-19), the outbreak of which occurred in the Wuhan city of China in December 2019 [1,2], has rapidly spread across the world. Although China, where the first cases of the disease were reported, is gradually recovering from this global pandemic, most countries are still being ravaged by this lethal virus with the region by region cases reported by WHO on 5 August 2020 as follows: American: 9,841,842, Europe: 3,451,556, South-East Asia: 2,299,433, Eastern Mediterranean: 1,585,458, Africa: 834,147, and Western Pacific: 341,165.

On 30 January 2020, the World Health Organization (WHO) declared COVID-19 a public health emergency of international concern and on 12 March, the WHO declared COVID-19 a pandemic. As of 5 August 2020, a total of 18,354,342 confirmed cases of infection were reported globally, with 697,147 confirmed deaths, and COVID-19 cases have been reported in 216 countries or areas of territories [3]. As there are currently no specific antiviral drugs to treat COVID-19, early detection and adherence to medical isolation become essential [4]. Confirmation of COVID-19 infection mainly depends on reverse transcription-polymerase chain reaction (RT-PCR) test using sputum, throat swabs, and other specimens [5,6]. COVID-19 is predominantly a disease of the respiratory system. The causative virus orchestrates deleterious inflammatory changes in the lung, which is its major target, but could also affect and damage other organs like the kidneys, heart, and liver [7,8].

Chest computed tomography (CT) is an important modality for detecting lung abnormalities [2]. CT plays a crucial role in the screening and management of suspected patients, and it is useful for both diagnosis and differential diagnosis, disease progression, and follow-up of COVID-19 patients [9]. Its morphological features change over time, with different presentations according to the phase and severity of lung infection. This phase is classified into four stages (early 0–4 days, progressive 5–8 days, peak 9–13 days, and absorption ≥ 14 days) according to time periods [10]. Chest CT can detect COVID-19 patients who have been symptomatic for more than three days, as more than 50% of patients imaged during the first 2 days following symptom onset may have normal CT findings [10]. Although it is very difficult to distinguish between CT manifestations of COVID-19 pneumonia from other viral influenza pneumonia due to overlap, CT examinations need to be combined with clinical indicators for comprehensive evaluation [11]. The most common chest CT manifestations of COVID-19 are bilateral and peripheral ground-glass opacities (GGO), consolidative pulmonary opacities which show sometimes rounded morphology, and peripheral lung distribution [10,12,13]. Studies have shown that chest CT has higher sensitivity than RT-PCR. Many suspected patients with abnormal CT findings initially gave false-negative results with RT-PCR test [14,15,16] prompting the procedure to be repeated multiple times, in some cases, for confirmation. This has, therefore, given credence to the assertion that CT scans can decrease the chance of false-negative results in the RT-PCR assay.

Recently, Kim et al. [17] performed a meta-analysis study to compare the performance of RT-PCR for COVID-19 patients and reported higher sensitivity compared to RT-PCR, and this sensitivity was affected by the distribution of disease severity, the proportion of patients with comorbidities, and the proportion of asymptomatic patients. Bao et al. [18] systematically reviewed the result of COVID-19 to report the most common imaging findings of CT. The former had limitations because it was limited to only a comparison of diagnostic accuracy chest CT and RT-PCR, while the latter did not classify the clinical symptoms into different groups. Moreover, other analysis related to the prevalence of bio-data information is absent, thus creating a considerable research gap in CT imaging of COVID-19 patients.

This systematic review and meta-analysis aim to quantitatively evaluate the prevalence of diagnostic performance of chest CT compared with RT-PCR, the prevalence of CT imaging morphological abnormalities, disease prevalence of different clinical symptoms, and other biodata information of COVID-19 patients.

## 2. Methods and Materials

This study was exempted by our institutional review board, and informed consent was not necessary.

### 2.1. Search Strategy and Study Selection

**Figure 1:**
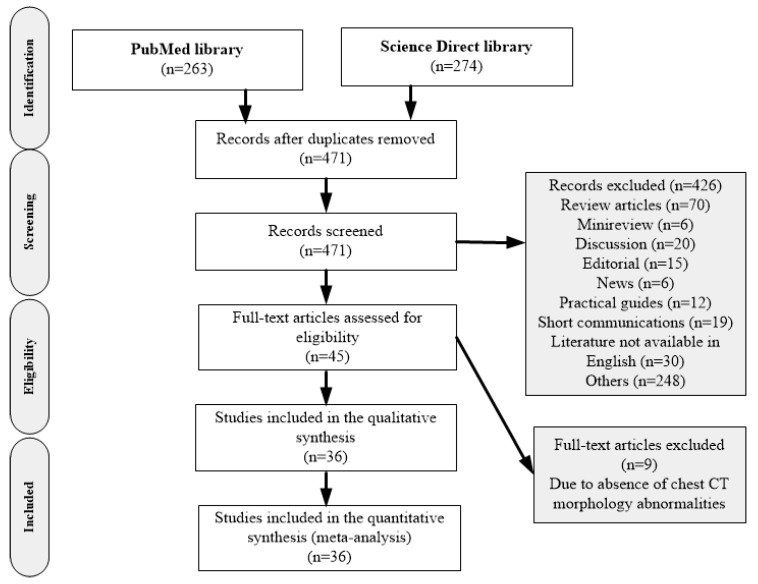
Study of inclusion and exclusion flowcharts adapted from the Preferred Reporting Items for Systematic Reviews and Meta-Analyses (PRISMA). n = number of literature and PRISMA = Preferred Reporting Items for Systematic Reviews and Meta-analyses, CT = Computed tomography.

This study followed the Preferred Reporting Items for Systematic Reviews and Meta-Analyses (PRISMA) reporting guidelines and was prospectively registered at PROSPERO (ID: CRD42020183067). We searched PubMed and Science Direct libraries from 1 January 2020 to 30 April 2020 for studies on COVID-19 that reported the diagnostic sensitivity of chest CT with these keywords: “computed tomography”, “COVID-19” and “Diagnosis”. The search strategy was designed by an experienced investigator with ten years of experience in both CT and MRI imaging (XY) and conducted independently by two authors (EOO and TEK). The literature search was unrestricted concerning the date, language, and region. Further, reference lists of reviews and conference proceedings were manually searched to identify gray literature (Figure 1). 

### 2.2. Study Inclusion and Exclusion

The included studies were Case studies, Cohort, Prospective, Retrospective and Observational studies, Randomized Controlled Trials, Pilot, Correlational Research Studies that fulfilled the following criteria: studies in which diagnostic performance measures and prevalence were extractable; and study populations of at least five patients with COVID-19. Exclusion criteria were review studies, case reports, or series with fewer than five patients, studies with lack of extractable data for sensitivity, and studies in which full text is not available in English.

### 2.3. Data Extraction

Two authors (JA and TEK) independently extracted the following data from each study: (1) Bio-data and patient characteristics (number of patients, age range, history of exposure, clinical symptoms, asymptomatic patients), (2) study characteristics (region of the study, disease prevalence, and the results of each diagnostic test of chest CT and RT-PCR assays). For the chest CT, the reference standard is RT-PCR, i.e., any positive results and all negative results from initial or repeated RT-PCR assays were regarded as disease positive and disease negative, respectively, while for the RT-PCR, all analyzed studies had repeated RT-PCR assays as the reference standard based on the eligibility criteria, (3) Chest CT morphology abnormalities and lesion distribution pattern and lung lobe location.

### 2.4. Study Quality Assessment

Study quality was independently assessed by two blinded reviewers (JA and TEK) with approximately five years’ experience in CT imaging, each utilizing the Quality Assessment of Diagnostic Accuracy Studies 2 (QUADAS-2) tool, which is recommended by the Cochrane collaboration for quality assessment of diagnostic test accuracy studies [19]. Study quality was rated across four domains for risk of bias assessment and across three domains for concerns regarding applicability to our review questions [19].

### 2.5. Statistical Analyses

In this systematic review and meta-analysis, we adopted double arcsine transformation for meta-analysis of prevalence which addresses both the problem of confidence limits outside the [0, 1] range and that of variance instability experienced when some proportions of the data extracted were too high or too low [20]. The double arcsine method formulation was given as:

(1) $$t=sin^{-1}{\left(\frac{n}{N+1}\right)}^{0.5}+sin^{-1}{\left(\frac{n+1}{N+1}\right)}^{0.5}$$

With its variance and back transformation to a proportion given by *Var*(*t*) and *p*, respectively.

(2) $$Var\left(t\right)=\frac{1}{N+0.5}$$

(3) $$p=\frac{1}{2}\left[1-sgn(\cos t)\sqrt{1-{\left(\sin t+\;\frac{\left(\sin t-\frac{1}{\sin t}\right)}{N}\right)}^{2}}\right]$$

where *N* is the population size, *n* is the number of people in the category, ‘*sgn*’ is the sign operator, *t* is the variance, and *p* is the back transformation.

Since both the conventional Fixed-effect model (FE) and Random-effect model (RE) can not address the problem of inverse variance, we applied the quality effect model (QE) proposed by Doi et al. [21] with study weight distribution using the relation below:

(4) $$\bar{Q}\;=\;\sum_{1}^{n}\left(\frac{\left(1\;-\;Q_{j}\right)\times w_{j}}{\sum_{1}^{n}w_{j}}\right)$$

where the index $\bar{Q}$ gives the extent to which distribution of the inverse variance weight has occurred when applying QE weights, the higher Q-index, the more likely that the QE estimator has been reduced beyond inverse variance heterogeneity (IVH) estimator. As Q index increases the confidence interval width around the pooled estimate. For this meta-analysis, the values of computed Q are given in Table A2 using designed signaling questions in Table A1 (Appendix A).

Statistical heterogeneity between studies was evaluated with Cochran’s Q test and the $I^{2}$ statistic [22]. For $I^{2}$ a value >50% was considered to have severe heterogeneity. Publication bias was evaluated by constructing a funnel plot and by Egger’s test [23]. All *p*-values were based on two-sided tests. A *p*-value < 0.05 was considered to represent statistical significance, while the statistical analyses were conducted using MetaXL version 5.3 (EpiGear International Pty Ltd., NC, Sunrise Beach, Queensland, Australia) [24].

## 3. Results

### 3.1. Study Selection

Detailed search procedures are summarized in Figure 1. In total, 539 studies published between 1 January and 30 April 2020 were identified by the electronic search strategy, and 471 studies remained after duplicates were excluded. Through title and abstract review, 426 publications were excluded. The full texts of the 45 identified articles were retrieved for detailed evaluation. Of these, 9 articles did not meet the inclusion criteria, and the remaining 36 independent studies were used in the current analysis.

### 3.2. Comparison of Diagnostic Performance of Chest CT with Morphological Abnormalities and RT-PCR

**Figure 2:**
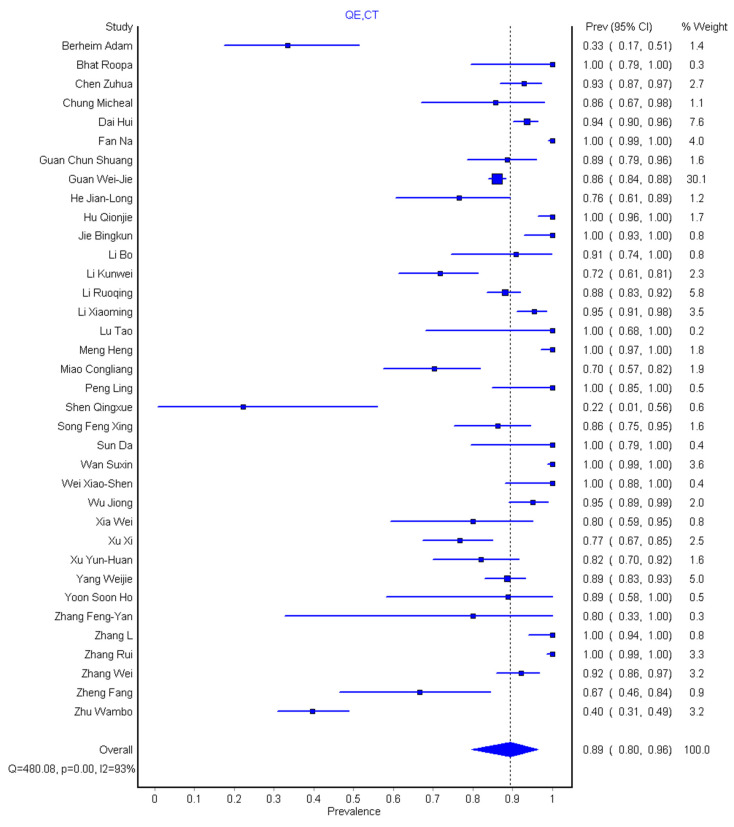
Forest plot of the pooled sensitivity of chest CT for detection of coronavirus disease 2019 (COVID-19) infection.

**Figure 3:**
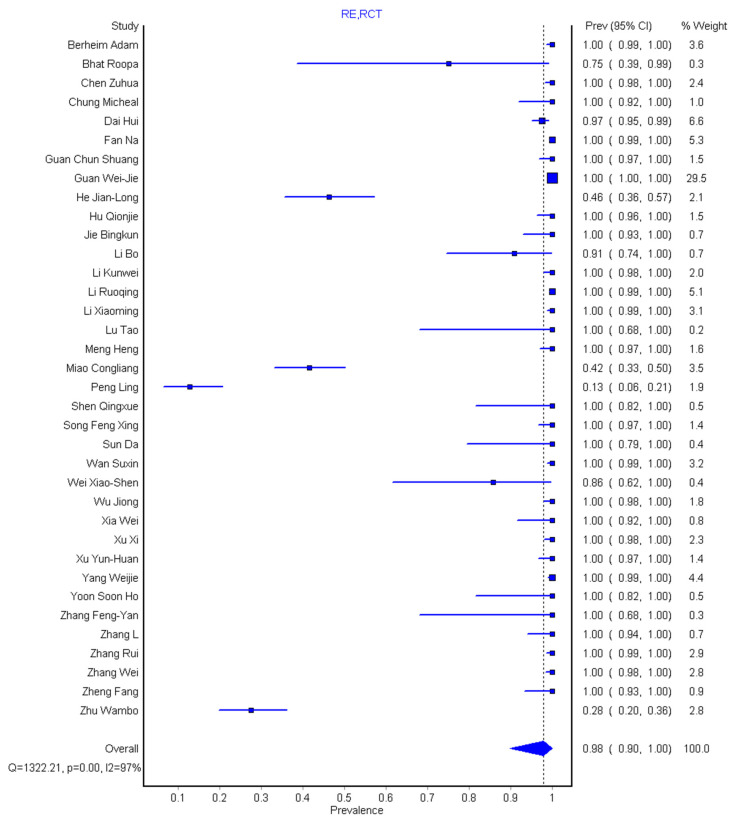
Forest plot of the pooled sensitivity studies for reverse transcriptase-polymerase chain reaction (RT-PCR).

In general, the 36 studies [1,2,12,13,25,26,27,28,29,30,31,32,33,34,35,36,37,38,39,40,41,42,43,44,45,46,47,48,49,50,51,52,53,54,55,56] had 3606 participants and 2842 (87%) of these had abnormal CT imaging features and fulfilled the requirement of the meta-analysis (Table 1). We categorized the CT morphological abnormalities into 10 groups as commonly found in the literature under study. The pooled sensitivity was 89% (95% CI: 80–96%) for the chest CT and 98% (95% CI: 90–100%) for the RT-PCR (Figure 2 and Figure 3). There was substantial heterogeneity for both the sensitivity of chest CT and RT-PCR. GGOs recorded the highest prevalence having present in 34/36 (94.4%) but absent only in 2/36 (5.6%) of the studies. Consolidations were present in 31/36 (86.1%) but 2/36 (5.6%) of the studies were found not applicable. Others are crazing-paving patterns 22/36 (61.1%), air bronchogram 16/36 (44.4%), adjacent pleural thickening 5/36 (13.9%), pleural effusion 16/36 (44.4%), pericardial effusion 2/36 (5.6%), lymphadenopathy 6/36 (16.7%), interlobular septa thickening 13/36 (36.1%), and other abnormalities (lymph node enlargement, bronchial dilation, centrilobular nodules, pulmonary nodules, cavitation, bronchiectasis) in 16/36 (44.4%) shown in Table 2.

**Figure 4:**
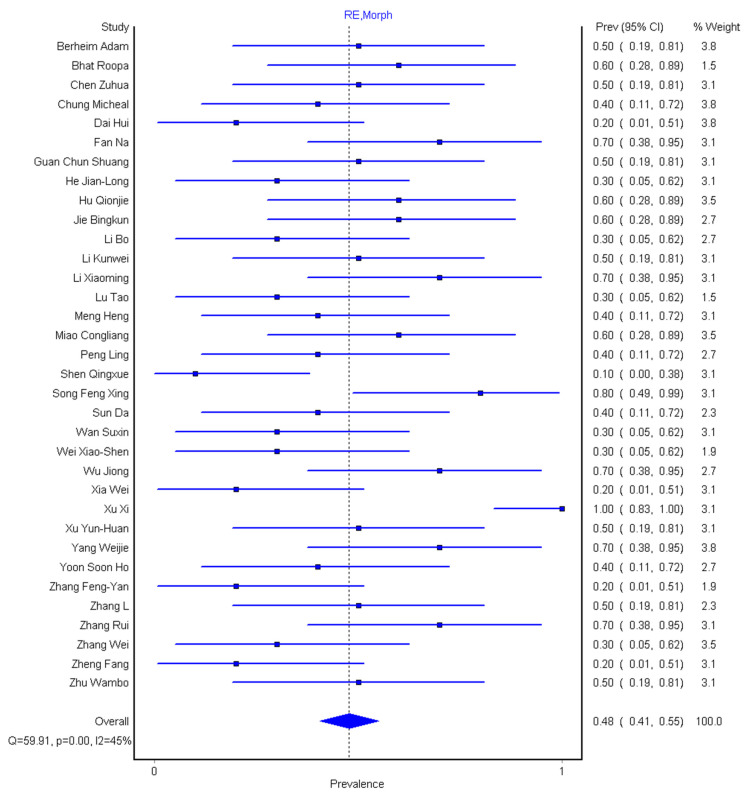
Forest plot of the pooled prevalence of morphological abnormalities among 34 patients.

The meta-analysis pooling was performed with 34 studies out of 36, and the pooled prevalence of all morphological abnormalities across the studies is 48% (95% CI: 41–55%), as shown in Figure 4.

### 3.3. Lesion Distribution and Lung Lobe Location

Three categories of lesion distribution were found across the 36 studies that fulfilled the requirement and were reviewed in the present study. These include bilateral, peripheral, and central distribution. Of the literature reviewed, 23/36 (63.9%) reported bilateral distribution and 4/36 (11.1%) were not applicable, 26/36 (72.2%) are peripheral of which 6 of 36 (16.7%) were not applicable, and 17/36 (47.2%) are central distribution but with 6/36 (16.7%) not applicable (Table 3). The meta-analysis pooling was performed with 30 studies out of 36, and the pooled prevalence of lesion distribution across the studies is 72% (95% CI: 62–80%), as shown in Table 4.

For the lung lobe location, five different locations of the lung lobe were found from the review as follows: Right upper lobe, right middle lobe, right lower lobe, left upper lobe, and left lower lobe. A total of 27/36 (75%) were found for the right upper lobe and 26/36 (72.2%) for the right middle lobe, right lower lobe, left upper, and left lower lobe, while 7/36 (19.4%) are not applicable (Table 3). Twenty-nine studies were used for pooling the meta-analysis and the pooled prevalence across all the lung lobe is 92% (95% CI: 84–97%). The result signifies that a significant proportion of patients had lesion distribution in each of the five lobes or in the combinations of two or more.

### 3.4. Exposure History and Patients’ Symptoms

**Figure 5:**
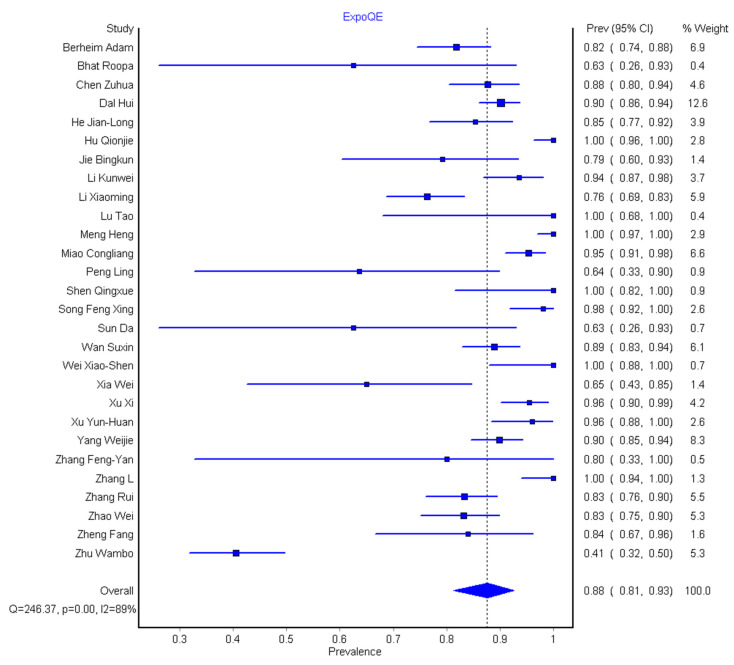
Forest plot of the studies for the pooled prevalence of exposure history for 28 included patients.

Of the 36 eligible studies, 2805/3606 (77.8%) had a history of exposure well documented. Out of these, 2387/2805 (85.1%) had contact with infected patients or had a recent travel history to a high-risk area with exposure history ranging from 4% to 100%, while 418/2805 (14.9%) had no contact with infected patients, which ranged from 0 to 59.1% (Table 1). The pooled prevalence with history of exposure was performed on the rest of 28 studies, with the pooled prevalence of 88% (95% CI: 81–93%) as shown in Figure 5. This result indicates that the majority of the COVID-19 patients had either direct or indirect contact with the infected patients.

**Figure 6:**
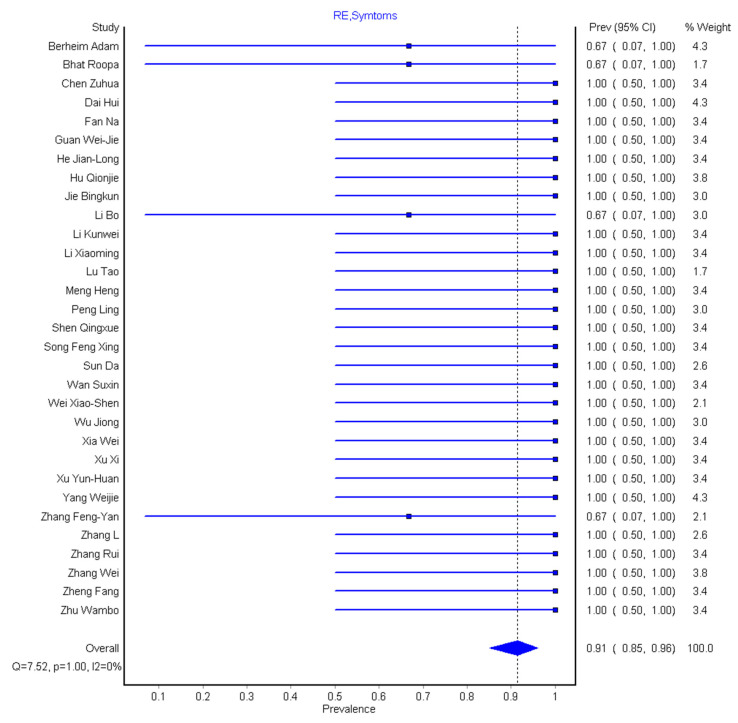
Forest plot of the pooled prevalence of grouped clinical symptoms (i.e., mild, moderate, and severe symptoms).

We grouped various clinical symptoms experienced by COVID-19 patients into four sub-sections: asymptomatic (without any clinical symptoms), mild symptoms (cough, vomit), moderate symptoms (cough, fever, headache, diarrhea), and severe symptoms (dyspnea, fever, myalgia, and failure of other vital organs due to the disease severity). The result shows that 6/36 (16.7%) are asymptomatic patients with 4/36 (11.1%) not eligible. Thirty-one of 36 (86.1%) present with mild symptoms, 32/36 (88.9%) with moderate symptoms, and 29/36 (80.6%) with severe symptoms (Table 1). We pooled the combined prevalence for all the clinical symptoms across the 32 eligible studies and this was found to be 91% (95% CI: 85–96%), as shown in Figure 6. The result indicates that few studies recorded cases of asymptomatic patients, while the majority of the studies recorded mild to severe symptoms.

### 3.5. Analysis of Patient Bio-Data Information

**Figure 7:**
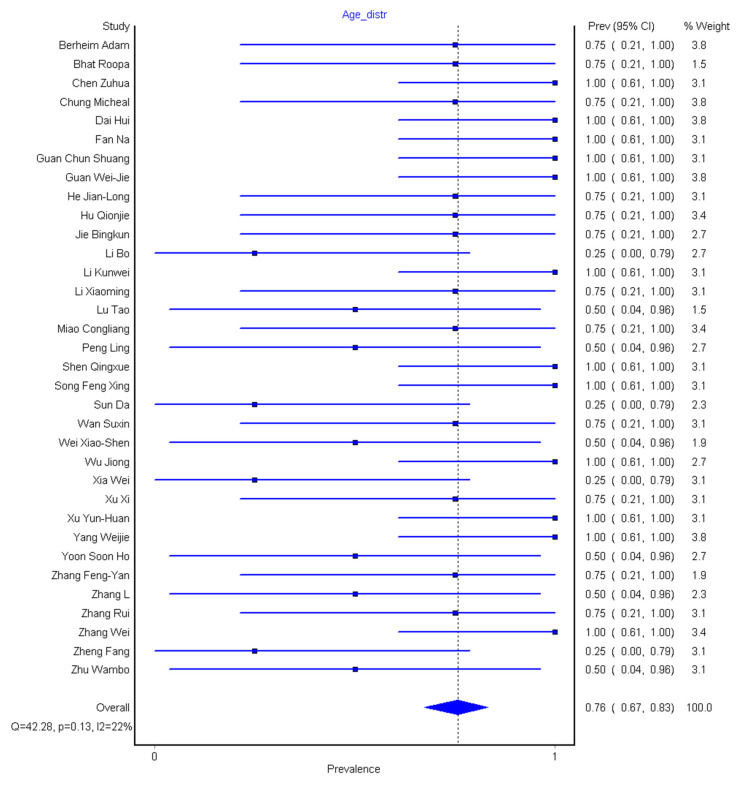
Forest plot of the prevalence of age distribution among patients, which include children, youth, adults, and elderly.

We grouped the age category into four: Children (0–17 years), youth (18–44 years), adults (45–59), and elderly (≥60) (Table 1). In the 36 eligible studies that had 3606 participants, the overall mean age of total participants is 42 years and ranged from 3 to 65 years. Out of these participants, 16/36 (14.4%) are children, 26/36 (72.2%) are youth, 30/36 (83.3%) are adults, and finally, 28/36 (77.8%) are elderly, while 2/36 do not have a documented age range. We pooled the prevalence of age across all the 34 studies and found the prevalence of age group distribution to be 76% (95% CI: 67–83%), as shown in Figure 7. This indicates that disease prevalence is widely distributed among all age groups, although the prevalence is slightly higher in the elderly and lower in children.

**Figure A1:**
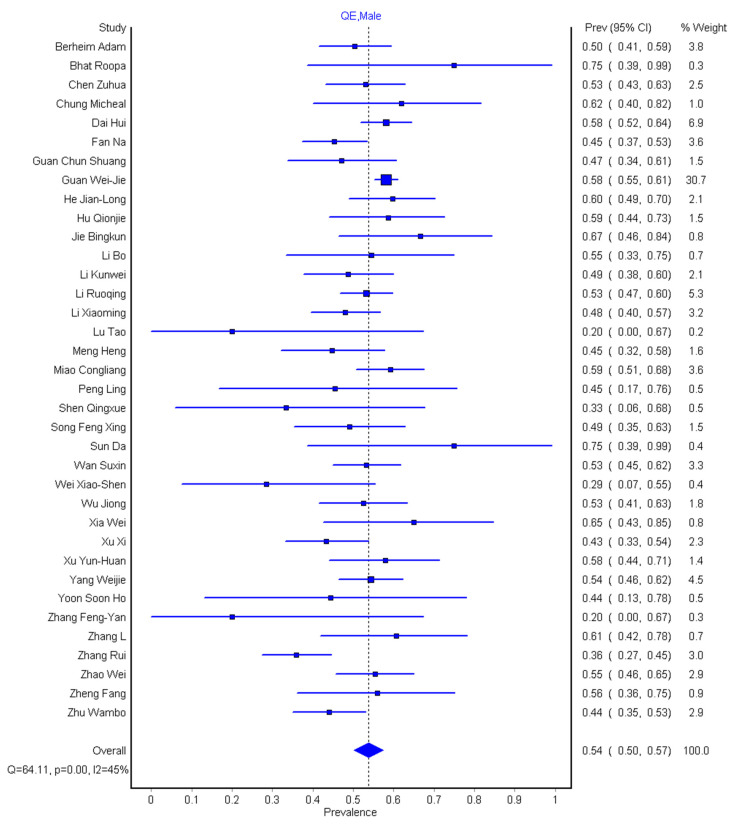
Forest plot of the pooled prevalence of male patients.

**Figure A2:**
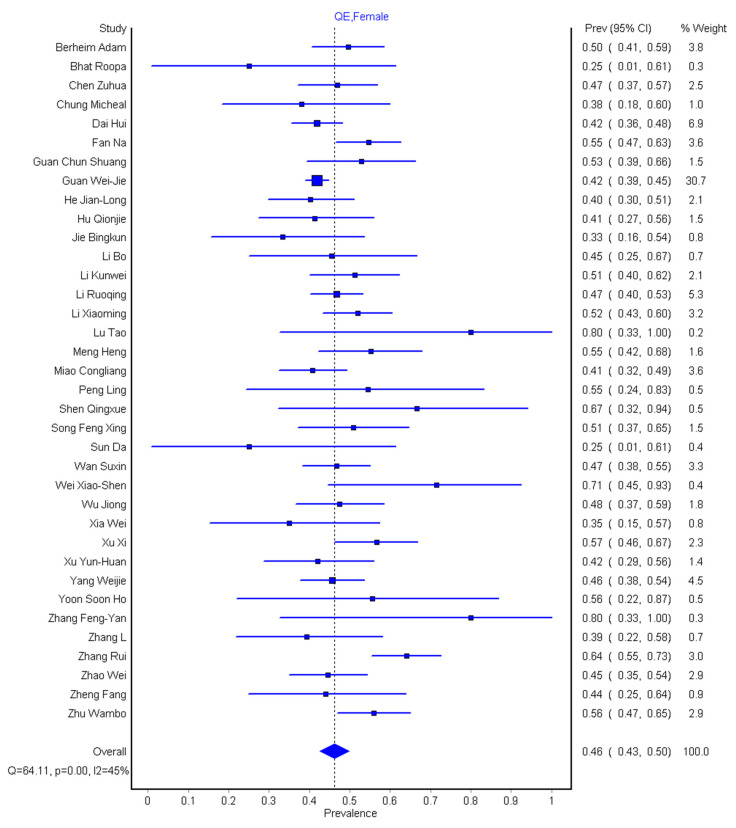
Forest plot of the pooled prevalence of female patients.

For the gender distribution, we pooled the prevalence of male and female participants separately across the 36 studies and the results show that males have 54% (95% CI: 50–57%) prevalence in a total population of 1934 and females have 46% (95% CI: 43–50%) prevalence in a total population of 1672. The result further indicates that the pooled prevalence of males is slightly higher than that of their female counterparts across the studies, as shown in Figure A1 and Figure A2 (Appendix A).

### 3.6. Quality Assessment

**Figure 8:**
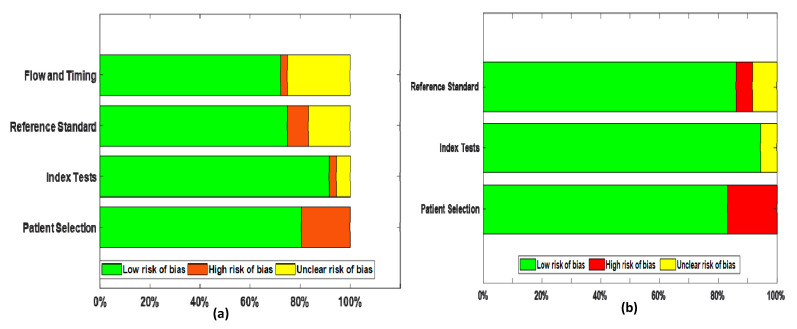
Stacked bar chart of QUADAS-2 domain versus the proportion of included studies for 36 studies (**a**) Risk of bias (**b**) Concerns regarding applicability. QUADAS-2 = Quality Assessment of Diagnostic Accuracy Studies 2.

All the included studies had a relatively low risk of bias in all domains (Figure 8), slightly above 70% in all four domains. Regarding patient selection, few studies were case studies that do not utilize a random process of patient selection, which might increase the risk of bias in that domain. Scanty information is available for few studies in Flow and Timing domain, thus causing an unclear risk of bias. Nevertheless, this paucity in detailed information did not raise any concerns regarding the applicability of the chest computed tomography.

### 3.7. Publication Bias

**Figure A3:**
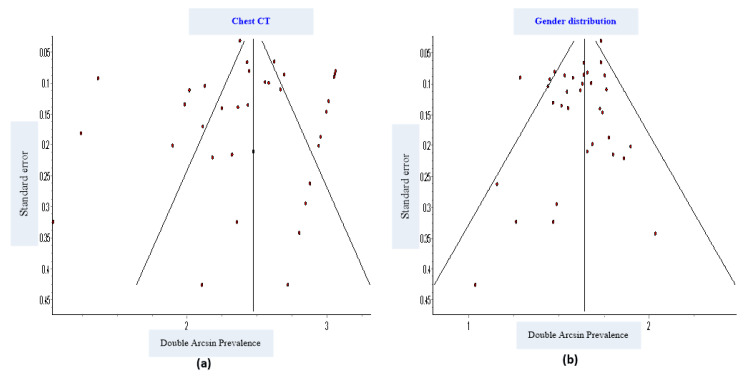
Funnel plots; the likelihood of publication bias was low for studies on (**a**) chest Computed tomography (CT) scans (**b**) Gender distribution (male and female).

The *p*-values for Egger’s regression symmetry test are shown in Table 4. The results showed that there was a low probability of publication bias in lesion distribution, patient clinical symptoms, and age distribution. Furthermore, the visual assessment of funnel plots demonstrated that the likelihood of publication bias was low for the aforementioned studies in Figure A3 (Appendix A).

## 4. Discussion

The result of systematic review and meta-analysis of 3606 participants shows that the pooled sensitivity was 89% (95% CI: 80–96%) for chest CT and 98% (95% CI: 90–100%) for RT-PCR. Our result is within the range of sensitivity (89.8–90.4)% given by Bao et al. [18] but gives a lower value compared to 94% sensitivity obtained by Kim et al. [17]. However, our RT-PCR pooled sensitivity showed a 10% increase over that of Kim et al. [17]. The slight reduction in pooled sensitivity of chest CT as supported by Berheim et al. [12] suggest that chest CT as a stand-alone procedure might not be so effective to diagnose patients with COVID-19, more so that there is an overlap of CT morphological abnormalities with other pneumonia caused by another virus as supported by Hani et al. [10]. The pooled prevalence of different CT abnormalities across the study was 48% (95% CI: 41–55%) and the pooled prevalence of the history of exposure for the 28 studies is 89% (95% CI: 83–94%), while that of age prevalence is 76% (95% CI: 67–83%) and prevalence of male to female is 54%(95% CI: 50–57%) to 46% (95% CI: 43–50%), respectively.

Ground-glass opacities (GGOs) recorded the highest CT morphological abnormalities as supported by different studies [26,27,28,29,30,31,32,33,34,35,36,37,38,39,40,41,42,43,44,45,46,47,48,49,50,51,52,53,54,55,56], whereas CT abnormalities like pericardial effusion, adjacent pleural thickening, and lymphadenopathy are scarcely reported [1,2,34,37,38,44,46,48,52]. The result of lobe location shows that a significant proportion of patients had lesions widely distributed in the five lobes or combinations of two or more.

In terms of exposure history, the majority of all suspected COVID-19 patients had a history of exposure by either direct or indirect contact. The result shows that about 17% of all COVID-19 patients are asymptomatic, while above 80% show mild, moderate, severe, or even a combination of either of the symptoms, which strongly suggest an interrelationship between mild, moderate, and severe clinical symptoms. Moreover, literature documentation revealed a higher prevalence for male patients compared to their female counterparts and this is supported by most of the studies in our analysis [1,2,12,13,28,29,30,31,32,34,38,45,46,48,49,52,54]. The results indicate that the disease prevalence is widely distributed among all age groups, even though it is slightly higher in the elderly and lower in children. The lower prevalence in children is not as a result of stronger immunity but rather because of relatively lesser exposure to infected persons, unlike the youth and adults. In contrast, the higher prevalence in the elderly may be as a result of higher exposure and a weak immune system. It has actually been observed that people with underlying health conditions and the elderly ones with reduced immunity are more likely to be at a higher risk for severe COVID-19 illness [57,58].

It should be noted that the computed value for Higgins (*I^2^*) indicated that there was substantial heterogeneity in chest CT sensitivity and history of exposure as widely supported by Kim et al. [17], while CT morphological abnormalities, gender distribution, age prevalence, and lung lobe location had moderate heterogeneity.

Our meta-analysis has several strengths. First, the pooled sensitivity was done across all ages and gender, which could serve as baseline studies across target population studies. Second, the studies were able to categorize the clinical symptoms into mild, moderate, and severe symptoms for easy identification. Third, different CT morphological abnormalities and lung lobe locations were extracted.

Nevertheless, several limitations exist. First, most of our studies had retrospective designs due to the current issue of the pandemic, as prospective diagnostic studies may not be feasible and also few numbers of included studies are case reports. Second, because of different models of CT scanners and radiologist experience in chest CT interpretation, reported chest CT morphological features may vary across each study. Third, since the mean age of the patient in each study is widely reported, it is challenging to classify results based on age group and vice versa.

In conclusion, this meta-analysis shows substantial heterogeneity in the sensitivity of both chest CT and RT-PCR while the latter possess higher overall sensitivity for the detection of COVID-19. GGOs represent the most significant CT morphological abnormality in patients and lesions assume bilateral, peripheral, and central distributions in some or all of five lung lobe locations. The reduction in the pooled sensitivity of chest CT in addition to the overlap of CT morphological abnormalities with pneumonia caused by another virus may hamper full implementation of chest CT as a standalone diagnostic tool for COVID-19. Reviewed cases are majorly symptomatic patients with mild to a horrendous degree of severity, and most patients have a history of either direct or indirect contact with infected persons. Across the studies, disease prevalence is widespread among all age groups but relatively higher in the elderly than in children and in males than in females.

## Figures and Tables

**Table 1:** Extraction table with biodata information, clinical symptoms of all eligible studies.

S/N	Study Type	Nationality	Patients No Abnormal/Normal	Gender M/F	Age Range					History of Exposure Yes/No (%)	Clinical Symptoms				PRT-RCT No. +ve/Total
					C	Y	A	E	Avg.		N	L	M	S	
1.	Berheim et al. [12]	China	10/30	61/60	−	+	+	+	45	82/18	−	+	+	−	121/121
2.	Bhat et al. [1]	U.S.	8/8	6/2	−	+	+	+	54.5	62.5/37.5	−	−	+	+	6/8
3.	Chen et al. [2]	China	91/98	52/46	+	+	+	+	43	87.8/12.2	−	+	+	+	98/98
4	Chung et al. [13]	China	18/21	13/8	−	+	+	+	51	NA/NA	NA	NA	NA	NA	21/21
5.	Dai et al. [25]	China	219/234	136/98	+	+	+	+	44.6	90/10	+	+	+	+	228/234
6.	Fan et al. [26]	China	150/150	68/82	+	+	+	+	56	NA/NA	+	+	+	+	228/234
7.	Guan et al. [27]	China	47/53	25/28	+	+	+	+	42	NA/NA	NA	NA	NA	NA	53/53
8.	Guan et al. [28]	China	840/975	639/460	+	+	+	+	47	NA/NA	+	+	+	+	1099/1099
9.	He et al. [29]	China	26/34	49/33	−	+	+	+	52	85/15	−	+	+	+	38/82
10.	Hu et al. [30]	China	46/46	27/19	−	+	+	+	39	100/0	−	+	+	+	46/46
11.	Jie et al. [31]	China	24/24	16/8	−	+	+	+	48.8	79.2/20.8	−	+	+	+	24/24
12.	Li et al. [32]	China	20/22	12/10	+	−	−	−	8	NA/NA	+	+	+	−	22/22
13.	Li et al. [33]	China	56/78	38/40	+	+	+	+	44.6	93.6/6.4	−	+	+	+	78/78
14.	Li et al. [34]	China	198/225	120/105	NA	NA	NA	NA	50	NA/NA	−	+	+	+	225/225
15.	Li et al. [35]	China	125/131	63/68	−	+	+	+	45	76/24	−	+	+	+	131/131
16.	Lu and Pu [36]	China	5/5	1/4	−	−	+	+	52.4	100/0	−	+	+	+	5/5
17.	Meng et al. [37]	China	58/58	26/32	NA	NA	NA	NA	42	100/0	−	+	+	+	58/58
18.	Miao et al. [38]	China	38/54	77/53	−	+	+	+	43	95/5	NA	NA	NA	NA	54/130
19.	Peng et al. [39]	China	11/11	5/6	−	−	+	+	40.7	63.6/36.4	−	+	+	+	11/86
20.	Shen et al. [40]	China	2/9	3/6	+	+	+	+	7.5	100/0	+	+	+	+	9/9
21.	Song et al. [41]	China	44/51	25/26	+	+	+	+	49	98/2	−	+	+	+	51/51
22.	Sun et al. [42]	China	8/8	6/2	+	−	−	−	6.7	63/37	−	+	+	+	8/8
23.	Wan et al. [43]	China	135/135	72/63	−	+	+	+	47	88.9/11.1	−	+	+	+	135/135
24.	Wei et al. [44]	China	14/14	4/10	−	+	+	−	36	100/0	+	+	+	+	12/14
25.	Wu et al. [45]	China	76/80	42/38	+	+	+	+	44	NA/NA	−	+	+	+	80/80
26.	Xia et al. [46]	China	16/20	13/7	+	−	−	−	NA	65/35	−	+	+	+	20/20
27.	Xu et al. [47]	China	69/90	39/51	−	+	+	+	50	96/4	−	+	+	+	90/90
28.	Xu et al. [48]	China	41/50	29/21	+	+	+	+	46.6	96/2	−	+	+	+	50/50
29.	Yang et al. [49]	China	132/149	81/68	+	+	+	+	45.11	89.9/10.1	−	+	+	+	149/149
30.	Yoon et al. [50]	Korea	8/9	4/5	−	−	+	+	54	NA/NA	NA	NA	NA	NA	9/9
31.	Zhang et al. [51]	China	4/5	1/4	−	+	+	+	39.6	80/20	−	+	+	−	5/5
32.	Zhang et al. [52]	China	28/28	17/11	−	−	+	+	65	100/0	−	+	+	+	28/28
33.	Zhang et al. [53]	China	120/120	43/77	−	+	+	+	45.1	83.2/16.8	−	+	+	+	120/120
34.	Zhao et al. [54]	China	93/101	56/45	+	+	+	+	44.5	83.2/16.8	−	+	+	+	101/101
35.	Zheng et al. [55]	China	16/24	14/11	+	−	−	−	3	84/16	−	+	+	+	25/25
36	Zhu et al. [56]	China	46/116	51/65	−	+	+	−	40	40.9/59.1	−	+	+	+	32/116

Note. (−) = absent, (+) = present, NA = Not applicable for age distribution: C = Children (0–17 years), Y = Youth (18–44), A = Adults (45–59 years), E = Elderly (≥60 years). Clinical symptoms: N = asymptomatic patients, L = mild symptoms (Cough, vomit), M = moderate symptoms (Cough, Fever, Headache, Diarrhea), S = severe symptoms (Dyspnea, fever, myalgia and vital organ failure).

**Table 2:** Chest computed tomography (CT) morphology abnormality findings of all the eligible studies.

		1	2	3	4	5	6	7	8	9	10
1.	Berheim et al. [12]	+	+	+	+	−	−	−	−	−	+
2.	Bhat et al. [1]	+	+	−	−	+	+	−	+	+	−
3.	Chen et al. [2]	+	+	+	−	−	+	−	−	+	−
4.	Chung et al. [13]	+	+	+	−	−	−	−	−	−	+
5.	Dai et al. [25]	−	−	−	+	−	−	−	−	−	+
6.	Fan et al. [26]	+	+	+	+	+	−	−	−	+	+
7.	Guan et al. [27]	+	+	+	+	−	−	−	−	−	+
8.	Guan et al. [28]	+	NA	NA	NA	NA	NA	NA	NA	NA	NA
9.	He et al. [29]	+	−	+	−	−	−	−	−	−	+
10.	Hu et al. [30]	+	+	−	+	−	+	−	−	+	+
11.	Jie et al. [31]	+	+	−	+	−	+	−	−	+	+
12.	Li et al. [32]	+	+	+	−	−	−	−	−	−	−
13.	Li et al. [33]	+	+	−	+	−	+	−	−	+	−
14.	Li et al. [34]	+	NA	NA	NA	NA	NA	NA	NA	NA	NA
15.	Li et al. [35]	+	+	+	+	+	+	−	−	+	−
16.	Lu and Pu [36]	+	+	+	−	−	−	−	−	−	−
17.	Meng et al. [37]	+	+	+	+	−	−	−	−	−	−
18.	Miao et al. [38]	+	+	+	+	−	+	−	+	−	−
19.	Peng et al. [39]	+	+	+	−	−	−	−	−	+	−
20.	Shen et al. [40]	+	−	−	−	−	−	−	−	−	−
21.	Song et al. [41]	+	+	−	+	−	+	+	+	+	+
22.	Sun et al. [42]	+	+	+	−	−	+	−	−	−	−
23.	Wan et al. [43]	+	+	−	−	−	+	−	−	−	−
24.	Wei et al. [44]	+	+	+	−	−	−	−	−	−	−
25.	Wu et al. [45]	+	+	+	−	+	+	−	−	+	+
26.	Xia et al. [46]	+	+	−	−	−	−	−	−	−	−
27.	Xu et al. [47]	+	+	+	+	+	+	+	+	+	+
28.	Xu et al. [48]	+	+	−	+	−	+	−	−	+	−
29.	Yang et al. [49]	+	+	+	+	−	+	−	+	−	+
30.	Yoon et al. [50]	+	+	+	+	−	−	−	−	−	−
31.	Zhang et al. [51]	+	+	−	−	−	−	−	−	−	−
32.	Zhang et al. [52]	+	+	+	−	−	−	−	−	+	+
33.	Zhang et al. [53]	+	+	+	+	−	+	−	+	−	+
34.	Zhao et al. [54]	+	+	−	−	−	−	−	−	−	+
35.	Zheng et al. [55]	−	+	+	−	−	−	−	−	−	−
36.	Zhu et al. [56]	+	+	+	−	−	+	−	−	−	+

Note. Chest CT = Chest Computed tomography. Note: (−) = absent, (+) = present, NA = Not applicable (1) = Ground-glass opacities (GGOs), (2) = Consolidation (3) = Crazy-paving pattern (4) = Air bronchogram (5) = Adjacent pleural thickening (6) = Pleural effusion (7) = Pericardial effusion (8) = Lymphadenopathy (9) = Interlobular septal thickening (10) = Others (lymph node enlargement, bronchial dilation, centrilobular nodules, pulmonary modules, cavitation, Bronchiectasis, intralesional or perilesional enlargement, fibrotic lesion, peri-pleural distribution, reticulation, fibromyalgia, bronchitis).

**Table 3:** Lesion distribution and location of chest computed tomography (CT) abnormality findings in all eligible studies.

S/N	Study Type	Lesion Distribution			Lung Lobe Location				
		1	2	3	1	2	3	4	5
1.	Berheim et al. [12]	+	−	−	+	+	+	+	+
2.	Bhat et al. [1]	+	+	−	+	+	−	−	−
3.	Chen et al. [2]	−	+	+	+	+	+	+	+
4.	Chung et al. [13]	+	+	−	+	+	+	+	+
5.	Dai et al. [25]	+	+	−	+	+	+	+	+
6.	Fan et al. [26]	+	−	−	+	+	+	+	+
7.	Guan et al. [27]	+	−	−	+	+	+	+	+
8.	Guan et al. [28]	NA	NA	NA	NA	NA	NA	NA	NA
9.	He et al. [29]	NA	NA	NA	NA	NA	NA	NA	NA
10.	Hu et al. [30]	−	+	+	+	+	+	+	+
11.	Jie et al. [31]	+	+	+	+	+	+	+	+
12.	Li et al. [32]	+	+	+	+	−	+	+	+
13.	Li et al. [33]	+	+	+	+	+	+	+	+
14.	Li et al. [34]	−	+	−	+	+	+	+	+
15.	Li et al. [35]	−	+	+	+	+	+	+	+
16.	Lu and Pu [36]	−	+	−	+	+	+	+	+
17.	Meng et al. [37]	+	+	+	+	+	+	+	+
18.	Miao et al. [38]	+	+	−	+	+	+	+	+
19.	Peng et al. [39]	−	+	−	+	+	+	+	+
20.	Shen et al. [40]	NA	NA	NA	NA	NA	NA	NA	NA
21	Song et al. [41]	+	+	+	+	+	+	+	+
22.	Sun et al. [42]	+	NA	NA	NA	NA	NA	NA	NA
23.	Wan et al. [43]	NA	NA	NA	NA	NA	NA	NA	NA
24.	Wei et al. [44]	−	+	−	−	−	−	−	−
25.	Wu et al. [45]	+	+	+	+	+	+	+	+
26.	Xia et al. [46]	+	+	+	+	+	+	+	+
27.	Xu et al. [47]	+	+	+	+	+	+	+	+
28.	Xu et al. [48]	+	+	+	+	+	+	+	+
29.	Yang et al. [49]	−	+	+	NA	NA	NA	NA	NA
30.	Yoon et al. [50]	−	+	+	+	+	+	+	+
31.	Zhang et al. [51]	+	+	−	+	+	+	+	+
32.	Zhang et al. [52]	+	+	−	+	+	+	+	+
33.	Zhang et al. [53]	+	+	+	+	+	+	+	+
34.	Zhao et al. [54]	+	+	+	+	+	+	+	+
35.	Zheng et al. [55]	+	NA	+	NA	NA	NA	NA	NA
36.	Zhu et al. [56]	+	NA	NA	NA	NA	NA	NA	NA

Note. (−) = absent, (+) = present; NA = Not applicable; for lesion distribution: 1 = Bilateral, 2 = Peripheral, 3 = Central; for lung lobe location: 1 = Right upper lobe, 2 = Right middle lobe, 3 = Right lower lobe, 4 = Left upper lobe, 5 = Left lower lobe.

**Table 4:** Summary of meta-regression analysis for diagnostic sensitivity of chest CT and pooled prevalence of other parameters.

Characteristics	Number of Included Studies	Pooled Estimates (%)	Heterogeneity		Publication Bias
			$I^{2}\;\mathrm{Statistics}\;(\%)$	Cochran Q-Test (%)	*p*-Value
Chest CT sensitivity	34	89 (80, 96)	92.7	480.1	0.000
RT-PCR sensitivity	34	98 (90, 100)	97	1322.2	0.000
CT morphological abnormalities prevalence	34	48 (41, 55)	44.9	59.9	0.003 *
Lesion distribution prevalence	30	72 (62, 80)	0.00	27.6	0.541
Lung lobe location prevalence	29	92(84, 97)	54.7	61.8	0.000
History of exposure prevalence	28	88 (81, 93)	89	246.4	0.000
Clinical symptoms prevalence	31	91 (85, 96)	0.000	7.5	1.000
Age prevalence	34	76 (67, 83)	21.9	42.3	0.129
**Gender distribution**					
*Male prevalence*	36	54 (50, 57)	45.4	64.1	0.002 *
*Female prevalence*	36	46 (43, 50)	45.4	64.1	0.003 *

Note. Number in parenthesis are 95% confidence interval, *p*-value is for the association between the variable and the magnitude of the effect size. A two-tailed *p*-value < 0.05 indicated statistical significance. * *p* < 0.05.

## Appendix A

**Table A1:** Health states Quality Scale Variables adopted from MetaXL.

S/N	Signaling Questions	Scale Variables
Q1	Were the target population and the observation period well defined?	Yes = 1 No = 0
Q2	Diagnostic criteria	Use of diagnostic system reported (DSM, ICD, RDC) = 1 Own system /symptoms described/no system/not specified = 0
Q3	Method of case ascertainment	Community survey/multiple institutions = 2 Inpatient/inpatients and outpatients/case registers = 1 Not specified = 0
Q4	Administration of measurement protocol	Administered interview = 3 Systematic case note review = 2 Chart diagnosis/case records = 1 Not specified = 0
Q5	Catchment Area	Broadly representative (national or multi-site survey) = 2 Small area/not representative (single community, single university) = 1 Convenience sampling/ other (primary care sample/treatment group) = 0
Q6	Prevalence measure	Point prevalence (e.g., one month) = 2 12-month prevalence = 1 Lifetime prevalence = 0

**Table A2:** Estimation of rescaled quality rank for 36 eligible studies.

S/N	Study Type	Q1	Q2	Q3	Q4	Q5	Q6	Total Score	$\mathrm{Quality}\;\mathrm{Rank}\;(Q_{i})$
1.	Berheim et al. [12]	1	1	2	2	2	2	10	1
2.	Bhat et al. [1]	0	0	1	1	0	2	4	0.4
3.	Chen et al. [2]	1	1	1	2	1	2	8	0.8
4	Chung et al. [13]	1	1	2	2	2	2	10	1
5.	Dai et al. [25]	1	1	2	2	2	2	10	1
6.	Fan et al. [26]	1	1	1	2	1	2	8	0.8
7.	Guan et al. [27]	1	1	1	2	1	2	8	0.8
8.	Guan et al. [28]	1	1	2	2	2	2	10	1
9.	He et al. [29]	1	1	1	2	1	2	8	0.8
10.	Hu et al. [30]	1	1	1	2	1	2	9	0.9
11.	Jie et al. [31]	1	1	1	2	0	2	7	0.7
12.	Li et al. [32]	1	1	1	2	0	2	7	0.7
13.	Li et al. [33]	1	1	1	2	1	2	8	0.8
14.	Li et al. [34]	1	1	1	2	1	2	8	0.8
15.	Li et al. [35]	1	1	1	2	1	2	8	0.8
16	Lu and Pu [36]	0	0	1	1	0	2	4	0.4
17.	Meng et al. [37]	1	1	1	2	1	2	8	0.8
18.	Miao et al. [38]	1	1	2	2	2	2	9	0.9
19.	Peng et al. [39]	1	0	1	2	1	2	7	0.7
20.	Shen et al. [40]	1	1	1	2	1	2	8	0.8
21.	Song et al. [41]	1	1	1	2	1	2	8	0.8
22.	Sun et al. [42]	1	0	1	2	0	2	6	0.6
23.	Wan et al. [43]	1	1	1	2	1	2	8	0.8
24.	Wei et al. [44]	1	0	1	1	0	2	5	0.5
25.	Wu et al. [45]	1	1	1	1	1	2	7	0.7
26.	Xia et al. [46]	1	1	1	2	1	2	8	0.8
27.	Xu et al. [47]	1	1	1	2	1	2	8	0.8
28.	Xu et al. [48]	1	1	1	2	1	2	8	0.8
29.	Yang et al. [49]	1	1	2	2	2	2	10	1
30.	Yoon et al. [50]	1	1	1	2	0	2	7	0.7
31.	Zhang et al. [51]	1	0	1	1	0	2	5	0.5
32.	Zhang et al. [52]	1	0	1	2	0	2	6	0.6
33.	Zhang et al. [53]	1	1	1	2	1	2	8	0.8
34.	Zhao et al. [54]	1	1	1	2	2	2	9	0.9
35.	Zheng et al. [55]	1	0	1	2	2	2	8	0.8
36.	Zhu et al. [56]	1	1	1	1	2	2	8	0.8

Note. *Q_i_* is known as rescaled quality rank in a meta-analysis and has a monotonic relationship to the bias. Its value is imputed for estimation of quality effect (QE) model and its value ranges between 0 and 1. Q1–Q2 represents the different signaling questions in Table A1.
